# Analysis of Antichiral Thermomechanical Metamaterials with Continuous Negative Thermal Expansion Properties

**DOI:** 10.3390/ma13092139

**Published:** 2020-05-06

**Authors:** Debajyoti Saha, Paul Glanville, Eduard G. Karpov

**Affiliations:** 1 Department Civil and Materials Engineering, University of Illinois, Chicago, IL 60607, USA; dsaha7@uic.edu; 2 Gas Technology Institute, Des Plaines, IL 60018, USA; Paul.Glanville@gastechnology.org

**Keywords:** thermomechanical metamaterial, mechanical metamaterial, negative thermal expansion, negative expansivity, architectured material, bimetal strip, metamaterial design

## Abstract

Negative thermal expansion is an interesting and appealing phenomenon for various scientific and engineering applications, while rarely occurring in natural materials. Here, using a universal antichiral metamaterial model with bimetal beams or strips, a generic theory has been developed to predict magnitude of the negative thermal expansion effect from model parameters. Thermal expansivity of the metamaterial is written as an explicit function of temperature and only three design parameters: relative node size, chirality angle, and a bimetal constant. Experimental measurements follow theoretical predictions well, where thermal expansivity in the range of negative 0.0006–0.0041 °C^−1^ has been seen.

## 1. Introduction and Material System Definition

Metamaterials is a term that defines modern engineered materials with extreme properties and functionalities that are not available in natural materials. Veselago in 1967 [1] proved theoretically that materials with both negative permeability and negative permittivity could demonstrate a range of unprecedented properties varying from light source attraction to flat lens focusing. Later, Pendry and Smith [2,3,4] expanded these concepts to advanced resolution imaging and wave guiding technology [5,6,7,8,9]. The term metamaterials, though, was only first used by Walser in 2001 [10]. The prefix *meta* stands for Greek *beyond* or *after*, implying availability of additional dimensions in the property space of the metamaterials when compared to the usual materials. A major advancement seen in more recent literature is a realization that the reverse or expanded properties can be realized in an “effective” manner, from material responses only to certain excitation frequencies. Thus, a key concept of frequency-dependent material property, varying from positive to negative or even complex values, has emerged and flourished. This contrasted with the original idea of a negative material property viewed in an objective manner, as a basic frequency-independent material constant [1] that is much more difficult to achieve in practice. In particular, the concept of negative refractive index has also been seen in wave mechanics and phononics, where it is associated with negative effective bulk modulus and negative effective mass density, observed at certain frequency ranges of an incident acoustical signal. Newly realized phenomena of shielding, bending, and focusing of sound waves propagating through materials with those reverse effective properties could serve for many interesting practical applications [11,12,13,14]. 

The notion of a *mechanical* metamaterial is the most recent and emerging in the field. The main objective of research in the area of mechanical metamaterials is to demonstrate materials with exotic mechanical properties, such as Poisson’s ratio, Young’s modulus, as well as bulk and shear moduli. The successes in the field of optics and acoustics, enabled by the material’s internal structure engineering, have guided the theory and application of mechanical metamaterials. A common approach shared by most authors in the field is to view the unusual mechanical properties as a result of a smart internal structure of the metamaterial on a unit cell level [15,16,17,18,19,20,21,22,23]. A proper engineering design then may lead to some extreme properties that are not available in the base materials used to fabricate those structures. For example, Kolpakov [17] and Lakes [18,19] described latticeworks and polyform foam structures with negative Poisson’s ratios [17] that would expand laterally when a longitudinal tensile force is applied. This type of nonconvex microstructure can be interesting for aerospace and marine application because of their light weight and good absorption properties [20,21]. Many interesting properties and behaviors are realized from *bistable* unit cell designs in periodic metamaterials, including highly efficient energy damping and trapping [22,23], negative stiffness [24], negative incremental compressibility [25,26,27,28], and extensibility [29,30]. Other studies of materials with engineered internal structure showed opportunities for a Saint-Venant’s edge effect reversal [31], strain energy control and redirection by demand [32,33,34], loss of reciprocity of materials deformation [35], and the negative thermal expansion phenomenon [36,37,38,39,40]. Harnessing these advanced behaviors could enable many exciting solutions in architecture, energy systems, manufacturing industry, transportation, and other areas. 

**Figure 1:**
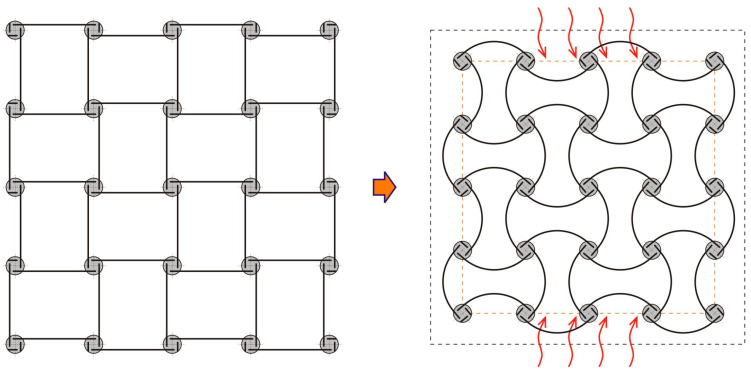
Antichiral metamaterial architecture, enabling negative (effective) thermal expansion. Continuous elastic thermal buckling of the bimetal strips, shown as solid lines, leads to an overall contraction of the material sample with temperature.

The main objective of this work is to provide a solid theoretical basis, backed up by experimental measurements, for a class of antichiral thermomechanical metamaterials with negative thermal expansion properties and continuous (nonsnapping) responses to thermal loads. A recent review of chiral metamaterial architectures is provided in reference [40]. One example from the literature, e.g., [38,40,41], is shown in Figure 1. Here, materials of the bimetal strips have a mismatch of their thermal expansion coefficients, leading to a continuous thermal bending of the strips and an overall size reduction of the material sample with temperature. A similar geometry was also discussed in the context of a continuous negative bulk modulus, which can be realized from a hydrostatic mechanical pressure applied to both sides of the composite strips whose materials have a mismatch of their elastic properties [41]. In the present paper, though, we focus on thermal responses of the Figure 1 type geometry. We also extend it to a generic antichiral geometry with arbitrary node shape/size, chirality angle and bimetal constant, and study dependence of the negative thermal expansion characteristics of these design parameters. 

**Figure 2:**
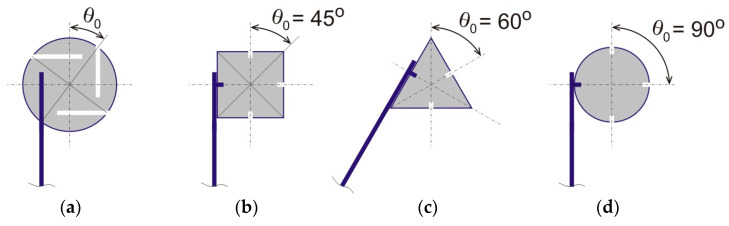
Definition of the chirality angle *θ*_0_, as a variable system parameter, and special cases of this angle for metamaterials with solid nodes of square, triangular, and circular shapes. (**a**) arbitrary $\theta_{0}$, (**b**) $\theta_{0}$ = 45°, (**c**) $\theta_{0}$ = 60°, (**d**) $\theta_{0}$ = 90°.

**Figure 3:**
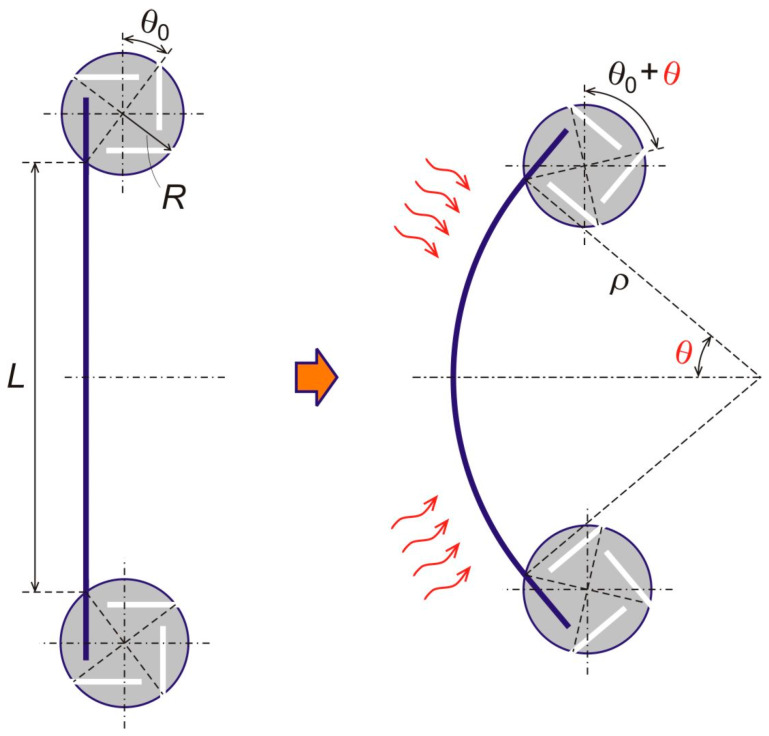
Three geometrical parameters of the metamaterial’s internal architecture ($\theta_{0}$, R, and L), and the node rotation angle (θ), as a single parameter to describe a thermally induced state of deformation. Here, ρ=L/θ is a radius of curvature of the thermally deformed strip.

Our generic metamaterial model is comprised of multiple bimetal strips of equal length and repeating solid nodes serving to connect the strips together. In practice, circular nodes with openings for strip insertion and fixture can be used to achieve an arbitrary *chirality* angle, $\theta_{0}$, see Figure 2a. Alternatively, the strips can be attached to side surfaces of polygonal of circular nodes for a specific value of the chirality angle (45°, 60°, or 90°), see Figure 2. The chirality angle is therefore a variable design parameter of this material system. It is generally defined as a minimal nonzero angle between a vertical symmetry axis of the material and a line passing through the node center and an endpoint of the free-standing part of a strip. Note that in all cases, portions of the bimetal strip in contact with nodes are assumed to be rigidly attached to the nodes in a nonslip manner. The remaining portion of the strips is free-standing, i.e., capable of a reversible mechanical buckling (bending) with heating or cooling (see drawings in Figure 2 and Figure 3).

Figure 3 drawing explains that three geometrical parameters—the chirality angle *θ*_0_, the node radius *R* (distance from a node center to an endpoint of the free-standing part of a bimetal strip), and length *L* of the free-standing parts of the bimetal strip—fully define the material’s internal architecture. We also introduce a single state parameter, which is the angle of rotation of the nodes *θ*, see Figure 3, to describe a state of deformation of the material due to heating. This state of deformation is defined by thermal bending of the bimetal strips upon uniform temperature change of the material, ∆*T*, with respect to an *initial* temperature at which all the strips are *straight*. Curvature of a thermally buckled strip is known to be uniform, so that the entire free standing strip takes a circular arch shape [42]. Therefore, from Figure 3,

(1) $$\theta =\frac{L}{2\rho }$$

where ρ is a uniform radius of curvature of the bimetal strips. It is interesting to note (e.g., from reference [42]) that dependence of the curvature on a temperature change is linear. Therefore, we suggest to introduce a system parameter, *a*, unique for a given bimetal strip, such that aΔT=1/ρ, and to write a linear constitutive relationship between the angle of deformation and temperature,

(2) $$\theta =\frac{L}{2\rho }=\frac{aL\Delta T}{2}$$

The coefficient *a* can be interpreted as a specific (per unit length) thermal bending coefficient of a bimetal strip, which gives an amount of uniform bending deformation (in radians) per one-degree temperature change. The dimensionality of *a* is $\left[L^{-1}T^{-1}\right]$, and its value depends on the cross-section geometry of the bimetal strip, elastic and thermal expansion coefficients of the two metals, and their joining fabrication method. Because of these multiple factors, the thermal bending coefficient, *a*, in Equation (2) should generally be found experimentally, using its physical meaning. More details are given Section 3.

## 2. Theoretical Analysis 

In this section, we discuss a quantitative analysis method, definitions of thermal expansion characteristics of the antichiral metamaterials, and predictions of their values from key structural parameters, accompanied by practical design recommendations.

Some basic quantities that will be used in the analysis are the following. According to Figure 3, an original distance between centers of two nodes, when the strips are straight, at ∆*T* = 0,

(3) $$l_{0}=L+2R\cos\theta_{0}$$

Then, a deformed distance between centers of two nodes, after application of heat to the system,

(4) $$l_{T}=\frac{L_{T}}{\theta }\sin\theta +2R_{T}\cos\left(\theta +\theta_{0}\right)$$

Here, $L_{T}$ is a changed length of the strip after temperature is applied, $R_{T}$ is a changed radius of the nodes due to the temperature, θ is a node rotation angle, and $\theta_{0}$ is a constant chirality angle as in Figure 2.

The values $L_{T}$ and $R_{T}$ can be written in a standard form using the usual coefficients of linear thermal expansion of the strip material, $\alpha_{s}$ (cumulative), and of the node material, $\alpha_{n}$,

(5) $$L_{T}=L\left(1+\alpha_{s}\Delta T\right)$$

(6) $$R_{T}=R\left(1+\alpha_{n}\Delta T\right)$$

These provide a distance between two nodes in a thermally deformed configuration,

(7) $$l_{T}=\left(1+\alpha_{s}\Delta T\right)\frac{L\sin\theta }{\theta }+2R\left(1+\alpha_{n}\Delta T\right)\cos\left(\theta +\theta_{0}\right),\;\theta =\frac{aL\Delta T}{2}$$

### 2.1. Thermal Strain Function

The quantities $l_{0}$ and $l_{T}$ given by Equations (3) and (7) represent an original length and a deformed length of a repeating unit cell of the metamaterial. Therefore, we can write the thermal strain as

(8) $$\varepsilon_{T}=\frac{l_{T}-l_{0}}{l_{0}}=\frac{2\theta R\left(1+\alpha_{n}\Delta T\right)\cos\left(\theta +\theta_{0}\right)+L\left(1+\alpha_{s}\Delta T\right)\sin\theta }{\theta \left(L+2R\cos\theta_{0}\right)}-1$$

The values of $\alpha_{n}$ and $\alpha_{s}$ are from $10^{-6}$ to $10^{-5}\;{{}^{\circ}C}^{-1}$ in metal alloys, and we may often encounter a situation when $\alpha_{s,n}\ll aL$ for the metamaterials discussed here. If $\alpha_{s,n}/aL<10^{-3}$, the usual thermal expansion will have a practically negligible effect on the thermal strain (8) behavior. In this case, internal structural parameters of the metamaterial will dominate its effective thermal expansion properties. Looking at Equation (8), we realize that the thermal strain depends only on a ratio R/L, rather than separately on R and L. Moreover, the angle θ depends only on a product aL, and not separately on a and L. Thus, the number of independent structural parameters is only three ($\theta_{0}$, r, and aL), see Table 1. 

A final form of the thermal strain, as a function of only independent structural parameters and temperature, reads

(9) $$\varepsilon_{T}=\frac{2\theta r\left(1+\alpha_{n}\Delta T\right)\cos\left(\theta +\theta_{0}\right)+\left(1+\alpha_{s}\Delta T\right)\sin\theta }{\theta \left(1+2r\cos\theta_{0}\right)}-1,\;\theta =\frac{aL\Delta T}{2}=\frac{A\Delta T}{2}$$

**Figure 4:**
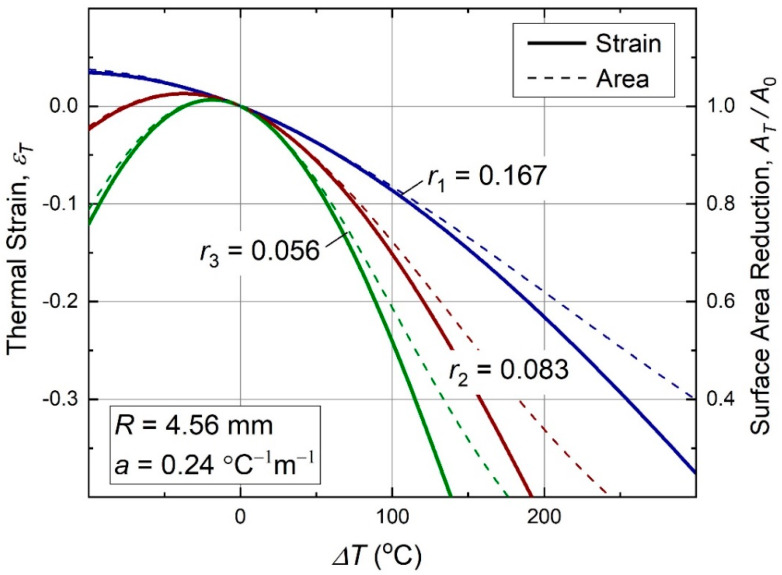
Behavior of the thermal strain (9) with temperature in antichiral thermomechanical metamaterials of Figure 1 type. The thermal strain is always negative at positive ∆*T*, and its overall behavior is nonlinear. Relative surface area reduction of a material sample, $A_{T}/A_{0}={\left(\varepsilon_{T}+1\right)}^{2}$, is shown with dash lines. For these and all further data plots, $\alpha_{s,n}\approx 10^{-3}aR/r$, so that the curve shapes are dominated by the metamaterial’s geometry, while the effect of natural thermal expansion is negligible.

In Figure 4, we show behavior of this function at some finite ratios *r*, and fixed a and R used in the experiments that will be described later in Section 3. Figure 4 also shows a relative surface area reduction of the metamaterial, $A_{T}/A_{0}={\left(\varepsilon_{T}+1\right)}^{2}$, where $A_{0}=l_{0}^{2}$ and $A_{T}=l_{T}^{2}$ are initial and reduced areas, respectively. This property could be interesting for autonomous safety systems applications of the present metamaterials, for example, serving to reduce throughput of pipes and vents with temperature.

### 2.2. Thermal Expansivity Function 

The thermal strain (9) is a nonlinear function of temperature, even at ΔT≈0. Therefore, we introduce a thermal expansivity function, $\alpha_{T}=\alpha_{T}\left(\Delta T\right)$, rather than a constant coefficient, as a derivative,

(10) $$\begin{array}{l} \alpha_{T}=\frac{d\varepsilon_{T}}{d\left(\Delta T\right)}\\ =\frac{2\left(1+\Delta T\alpha_{s}\right)\theta \cos\theta +4r\Delta T\alpha_{n}\theta \cos\left(\theta +\theta_{0}\right)-2\sin\theta -4r\left(1+\Delta T\alpha_{n}\right)\theta^{2}\sin\left(\theta +\theta_{0}\right)}{2\Delta T\theta \left(1+2r\cos\theta_{0}\right)} \end{array}$$

**Figure 5:**
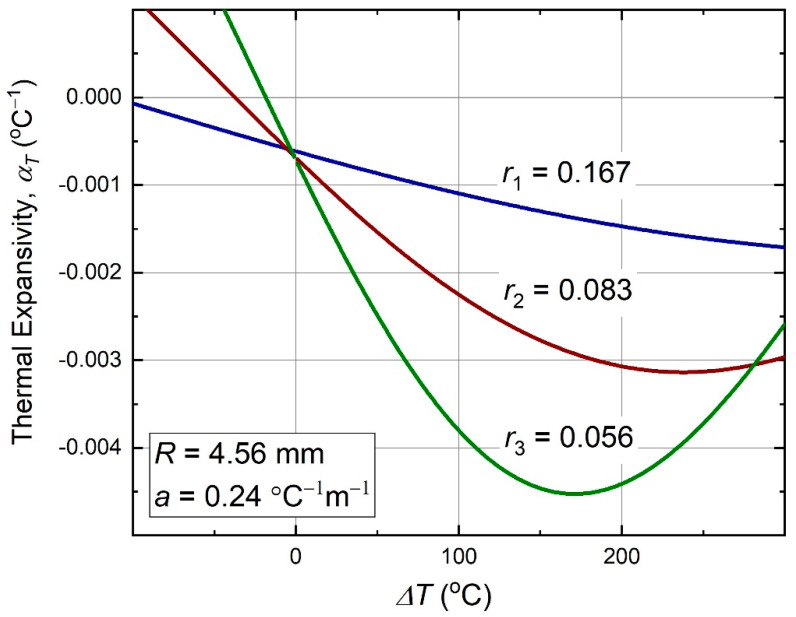
Thermal expansivity (10) of the antichiral metamaterial as function of temperature.

Behavior of this function with temperature is shown in Figure 5. As can be seen, it can be nonmonotonous, because of the sine and cosine functions involvement in the temperature dependence. Also, the negative thermal expansivity is better pronounced at higher nodal size ratios, r, which occur in the denominator of the Equation (10). 

### 2.3. Thermal Hyperexpansivity

A derivative of the thermal expansivity function (10) with respect to temperature can be referred to as thermal hyperexpansivity,

(11) $$\begin{array}{l} \alpha_{T}^{\prime }=\frac{d\alpha_{T}}{d\left(\Delta T\right)}\\ =-\frac{2\theta \cos\theta +2r\left(1+\Delta T\alpha_{n}\right)\theta^{3}\cos\left(\theta +\theta_{0}\right)-2\sin\theta +\theta^{2}\sin\theta +\Delta T\theta^{2}\left(\alpha_{s}\sin\theta +4r\alpha_{n}\sin\left(\theta +\theta_{0}\right)\right)}{{\left(\Delta T\right)}^{2}\theta \left(1+2r\cos\theta_{0}\right)} \end{array}$$

**Figure 6:**
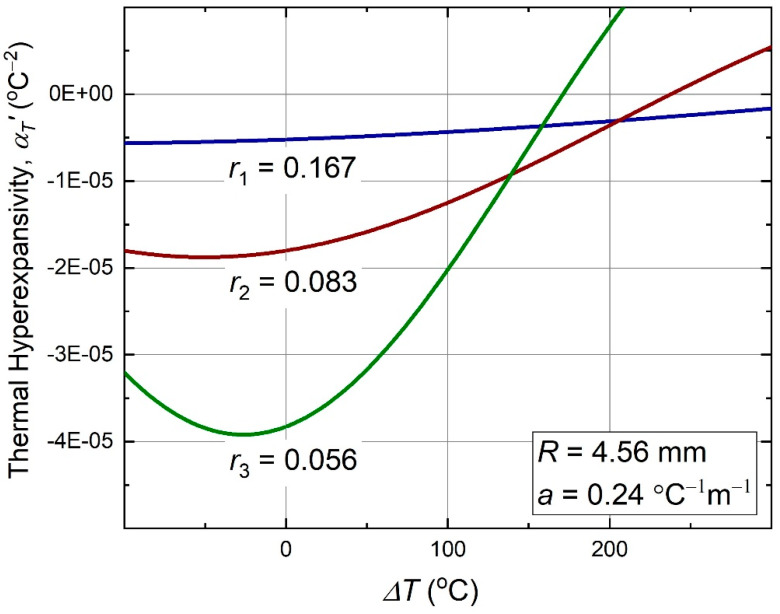
Thermal hyperexpansivity (11) of the antichiral metamaterial as function of temperature.

Plots of this function versus temperature, for the same three nodal size ratios, as in the previous plots of $\varepsilon_{T}$ and $\alpha_{T}$, are shown in Figure 6.

### 2.4. Initial Thermal Expansivity and Hyperexpansivity

Thermal expansivity of the antichiral metamaterial has a negative slope versus temperature at all combinations of the system parameters (see Figure 5 for an illustration) and a temperature increase further enhances magnitude of the negative thermal expansion effect. Therefore, for many practical purposes, it is interesting to study dependence of the initial (at ΔT≈0) values of the functions $\alpha_{T}$ and $\alpha_{T}^{\prime }$ on the system parameters. A combination of parameters at which these functions attain maximal possible (by modulus) values could be viewed as recommendations for a practical material design. 

If we employ a single parameter for the usual thermal expansion, $\alpha_{s}=\alpha_{n}$, recall that θ=aLΔT/2 and apply a power series decomposition of the thermal strain (9) at ΔT=0 up to a quadratic term,

(12) $$\begin{array}{ll} \varepsilon_{T}\approx \alpha_{0}\Delta T+ & \alpha_{0}^{\prime }\frac{{\left(\Delta T\right)}^{2}}{2}\\ & \;=\frac{\alpha_{s}\left(1+2r\cos\theta_{0}\right)-aLr\sin\theta_{0}}{1+2r\cos\theta_{0}}\Delta T\\ & \;-\frac{\left(24\alpha_{s}r\sin\theta_{0}+aL+6aLr\cos\theta_{0}\right)aL}{12+24r\cos\theta_{0}}\frac{{\left(\Delta T\right)}^{2}}{2} \end{array}$$

we may interpret the coefficient $\alpha_{0}$ at the linear term as an initial thermal expansivity (at ΔT≈0). This characteristic can be written in an interesting shorter form, a sum of the natural thermal expansion ($\alpha_{s}$), and a term depending only on the architectural design parameters ($\theta_{0}$, r and aL):

(13) $$\alpha_{0}=\alpha_{s}-\frac{raL\sin\theta_{0}}{1+2r\cos\theta_{0}}$$

**Figure 7:**
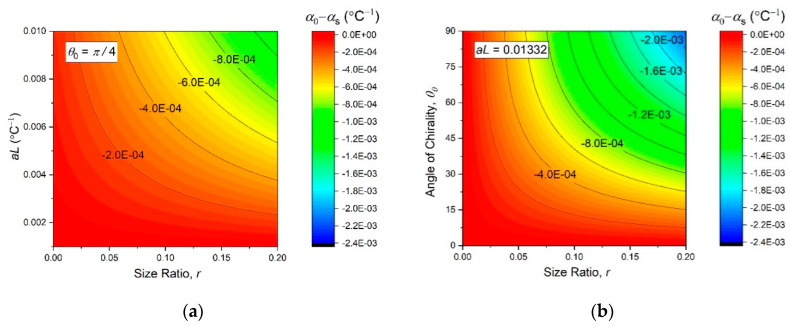
Contour plots of the initial thermal expansivity (13), as function of the independent design parameters ($\theta_{0}$, r, and aL).

Obviously, the first term can be ignored, if $\alpha_{s}\ll aL$. A practical range of the chirality angle is from 0^o^ to 90°, because values higher than 90° would require practically impossible connections to accommodate overlapping bimetal strips. We assumed some fixed values of $\theta_{0}$ and aL, as were later used in the experiments, and plotted $\alpha_{0}$ onto the contour maps of Figure 7. They reveal a monotonous dependence of $\alpha_{0}$ on all the independent structural parameters of the metamaterial, so that a greater negative thermal expansion effect at ΔT≈0 should generally be expected at larger values of $\theta_{0}$, r, and aL. 

**Figure 8:**
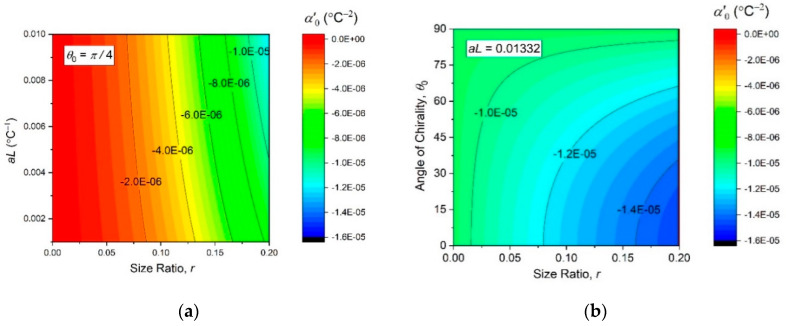
Contour plots of the initial thermal hyperexpansivity (14) as function of the independent design parameters ($\theta_{0}$, r, and aL).

From the power series expansion of the thermal strain (12), we may also define an initial hyperexpansivity of the antichiral metamaterial (at ΔT≈0 and $\alpha_{s}\ll aL$),

(14) $$\alpha_{0}^{\prime }=-\frac{aL\left(aL+6aLr\cos\theta_{0}\right)}{12+24r\cos\theta_{0}}$$

which is also monotonous with $\theta_{0}$, r, and aL, although a greater hyperexpansivity should be expected at smaller chirality angles, see Figure 8.

### 2.5. Maximal Rotation Angle -and Maximal Temperature

**Figure 9:**
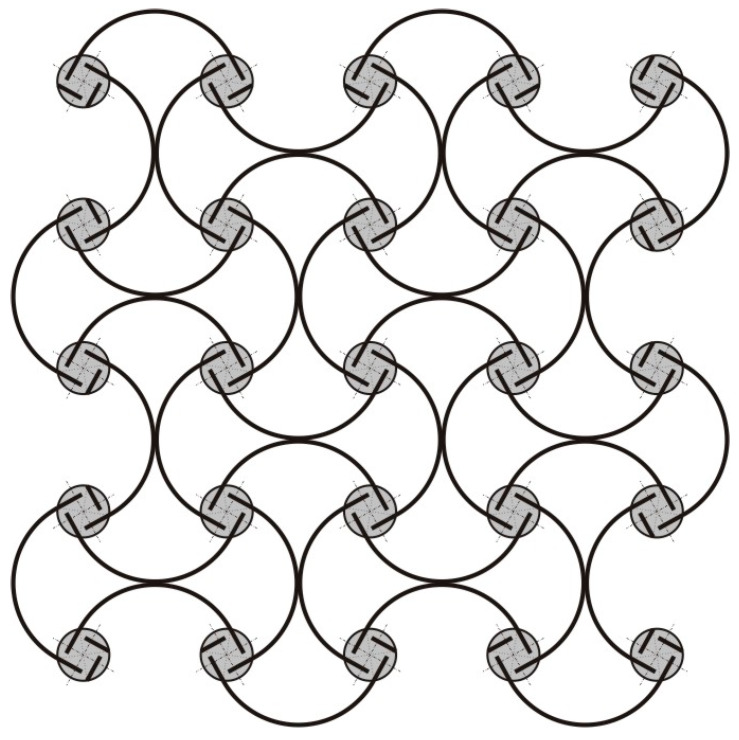
Occurrence of a maximal angle of nodal rotation, $\theta_{m}$, in the antichiral thermomechanical metamaterial of Figure 1 type.

Continuous thermal deformation in the antichiral metamaterials discussed here might be restrained in practice, because of a possible limiting configuration depicted in Figure 9. Here, two opposite bimetal strips in a unit cell encounter each other due to an excessive thermal deformation. This situation is unique for a particular metamaterial design $\left\{\theta_{0},\;r,\;aL\right\}$, and it is described by a nodal rotation angle, $\theta_{m}$, a maximal angle of applicability of the theory discussed here.

This angle can be determined from a condition that the deformed distance (7) between two nodes is equal to a double distance between the middle arch point of a bimetal strip, attached to these nodes, and the base line passing through the centers of these nodes. The usual thermal expansion has a negligible effect on the maximal angle value, when $\alpha_{s},\alpha_{n}\ll aL$. These to lead a maximal angle condition in the form,

(15) $$\frac{\sin\theta_{m}}{\theta_{m}}+2r\cos\left(\theta_{m}+\theta_{0}\right)=\frac{1-\cos\theta_{m}}{\theta_{m}}+2r\sin\left(\theta_{m}+\theta_{0}\right)$$

The corresponding maximal operational temperature of the metamaterial is

(16) $$\Delta T_{m}=\frac{2\theta_{m}}{aL}$$

**Figure 10:**
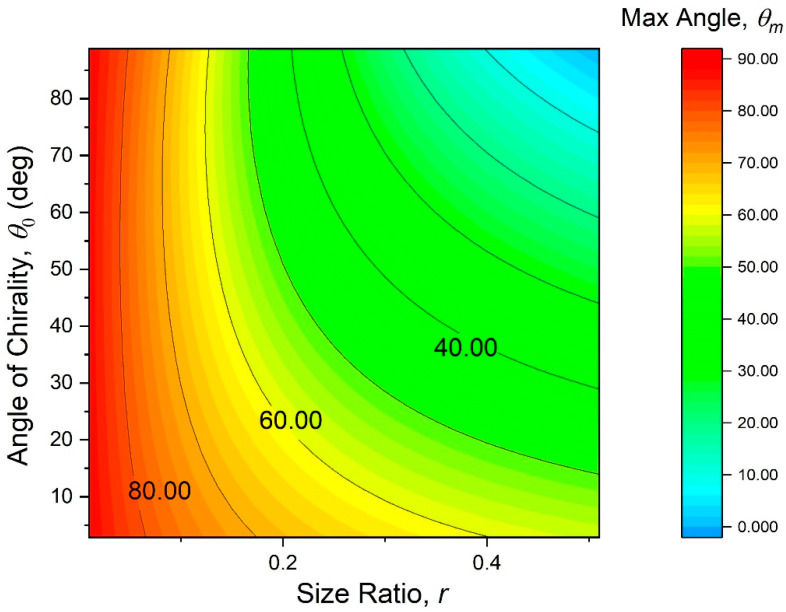
Contour map of the maximal allowed nodal rotation angle, $\theta_{m}$. The corresponding maximal operational temperature of the metamaterial is $\Delta T_{m}=2\theta_{m}\left(\mathrm{rad}\right)/aL$.

The transcendental Equation (15) is not solvable in a closed form for $\theta_{m}$. We solved is numerically and mapped the solution on the Figure 10 contour plot. According to this mapping, lower node size ratios maximize the critical angle $\theta_{m}$. At moderate node size ratios of 0.05–0.15, designs with chirality angles between 50–70° will lead to slightly lower values of $\theta_{m}$ than designs with other chirality angles.

## 3. Experimental Validation

For the metamaterials prototyping, a bimetal strip stock (SBC 721-112) by Shivalik Bimetal Controls Ltd has been utilized. Index 721 stands for an alloy of 72% Mn, 10% Ni, and 18% Cu, and 112 stands for the Invar alloy of 36% Ni and 64% Fe. Since the thermal bending coefficient in (2) is not a standard characteristic, we determined it experimentally. The follwoing value was found and used in Section 2 plots, when needed,

(17) $$a=0.24\pm 0.01\;\frac{\mathrm{rad}}{m\cdot {}^{\circ}C}$$

**Figure 11:**
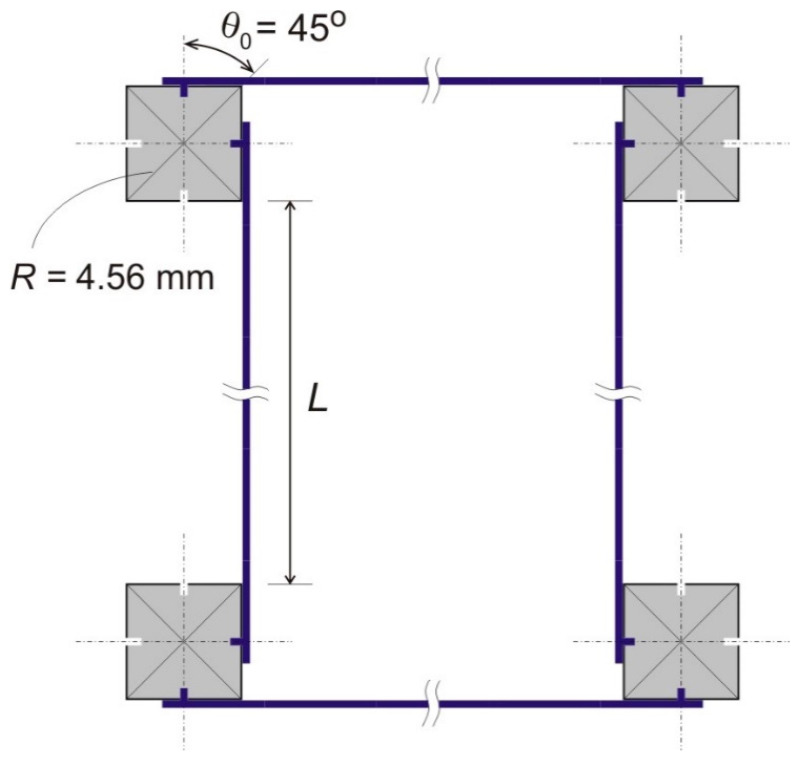
Schematic diagram of a representative unit cell in fabricated samples. Square nodes imply a chirality angle, $\theta_{0}=45{}^{\circ}$. Three different values of the length *L* were utilized.

**Figure 12:**
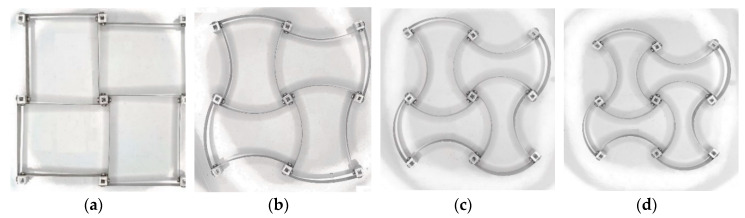
Recorded deformed shapes of a sample with *L* = 54.9 mm (refer to Figure 11) at four different temperatures ΔT: (**a**) 0 °C, (**b**) 65 °C, (**c**), 85 °C (**d**), 136 °C.

Assembled samples had a nondeformed geometry with square nodes depicted in Figure 11, where the chirality angle, $\theta_{0}=45{}^{\circ}$, and an equivalent nodal radius, R=4.56 mm. Samples with three different values of the free standing bimetal strip length were fabricated: *L*_1_ = 27.3 mm, *L*_2_ = 54.9 mm, and *L*_3_ = 81.4 mm. These correspond to the node size ratios *r*_1_ = 0.167, *r*_2_ = 0.083, and *r*_3_ = 0.056. Other parameters (*a*, $\theta_{0}$, *R*) were identical in all samples. The samples were heated in a mini environmental chamber to a known uniform temperature ∆*T*, and their deformed geometry was captured through a clear window with a high resolution camera. A digital image processing procedure was applied to determine a change of nodal distance with temperature, interpreted in terms of the thermal strain, $\varepsilon_{T}=\left(l-l_{0}\right)/l_{0}$. Some of the captured deformed shapes are shown in Figure 12.

**Figure 13:**
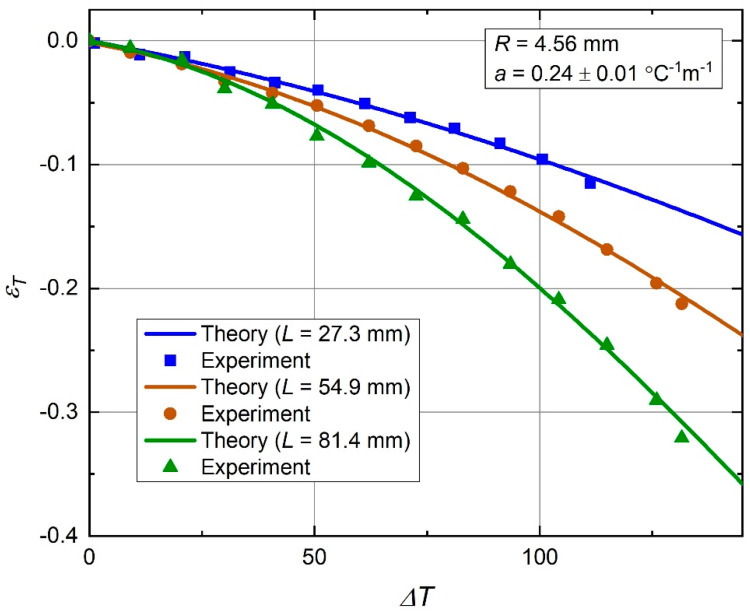
Behavior of the thermal strain ($\varepsilon_{T}$) with temperature: present theory and experiment.

The measured thermal strain values matched the theoretical curves well, as can be seen from the Figure 13 plot. Given a 4% uncertainty in the *a*-value measurement, Equation (17), plus an estimated ±0.01 systematic thermal strain error due to geometrical imperfections of the samples, this match can be considered very good. This proves validity of the theoretical analysis approach discussed in Section 2.1, Section 2.2, Section 2.3, Section 2.4 and Section 2.5. The observed thermal strain dependence on temperature corresponds to a thermal expansivity, whose values can be best seen from the earlier Figure 5 plot. For the temperature range of ΔT from 0 °C to 130 °C, it is in the range of negative 0.0006–0.0041 °C^−1^. The initial thermal expansivity (at ΔT≈0 °C) is the range of negative 0.0006–0.0007 °C^−1^, depending on the value *L*.

## 4. Conclusions

Negative thermal expansion phenomenon is interesting and appealing for various scientific and engineering applications; however, it rarely occurs in natural materials. In this paper, we have discussed a universal antichiral thermomechanical metamaterial model with bimetal beams or strips connected at solid nodes. A theoretical analysis approach has been developed to write thermal expansivity of the metamaterial as an explicit function of temperature and only three design parameters: relative node size, chirality angle, and a bimetal constant. Experimental measurements follow the theoretical predictions well, where a thermal expansivity in the range of negative 0.0006–0.0041 °C^−1^ has been seen.

In the future, the limit case configuration depicted in Figure 9 could be considered as an initial room temperature architecture, where the bimetal strips are joined to each other at their middle points. This will possibly lead to a lower negative thermal expansion effect, but will enhance mechanical properties of the metamaterial.

## Figures and Tables

**Table 1:** Independent structural parameters of the antichiral metamaterial.

Chirality Angle (rad)	Node Size Ratio	Thermal Bending Coefficient (rad/°C)
$\theta_{0}$	$r=\frac{R}{L}$	A=aL
